# Improved Electrical Properties of EHD Jet-Patterned MoS_2_ Thin-Film Transistors with Printed Ag Electrodes on a High-k Dielectric

**DOI:** 10.3390/nano13010194

**Published:** 2023-01-01

**Authors:** Thi Thu Thuy Can, Woon-Seop Choi

**Affiliations:** School of Electronics and Display Engineering, Hoseo University, Asan 31499, Republic of Korea

**Keywords:** MoS_2_, Ag electrode, EHD jet printing, high-k dielectric, thin-film transistors

## Abstract

Electrohydrodynamic (EHD) jet printing is known as a versatile method to print a wide viscosity range of materials that are impossible to print by conventional inkjet printing. Hence, with the understanding of the benefits of EHD jet printing, solution-based MoS_2_ and a high-viscosity Ag paste were EHD jet-printed for electronic applications in this work. In particular, printed MoS_2_ TFTs with a patterned Ag source and drain were successfully fabricated with low-k silica (SiO_2_) and high-k alumina (Al_2_O_3_) gate dielectrics, respectively. Eventually, the devices based on Al_2_O_3_ exhibited much better electrical properties compared to the ones based on SiO_2_. Interestingly, an improvement of around one order of magnitude in hysteresis was achieved for devices after changing the gate insulator from SiO_2_ to Al_2_O_3_. In effect, the results of this work for the printed MoS_2_ and the printed Ag source and drains for TFTs demonstrate a new approach for jet printing in the fabrication of electronic devices.

## 1. Introduction

Graphene, the first material in the two-dimensional (2D) family of materials, has been used in a large number of scientific applications due to its superior and novel properties, e.g., mechanical, thermal, electrical, optical, etc. [1]. Likewise, as an emerging candidate in the crowd of 2D materials, transition metal dichalcogenides (TMDs) have drawn much attention due to their sizeable bandgap, which is definitely better than the unfavorable zero bandgap of graphene [2]. In particular, molybdenum disulfide (MoS_2_) is one of the most studied TMDs, with diverse applications [3,4] because of its tremendously high intrinsic electron mobility and indirect-to-direct bandgap from 1.2 eV in bulk to 2.0 eV in the monolayer—especially in thin-film transistor device applications [5].

Researchers have devoted considerable efforts to synthesizing high-quality MoS_2_ with a controllable number of layers, playing a significant role in the fundamental research and application explorations involving this material. Generally, a variety of methods have been proposed to produce 2D TMD materials, including mechanical/chemical exfoliation [6,7], chemical vapor deposition (CVD) [8], wet-chemical based methods [9], and so on. Even though the growth of atomically thin TMD films via the CVD method is one of the most popular ways of producing these films, controlling the respective concentrations of the precursors precisely during the growth process is still challenging. Next, exfoliation methods, despite their simple and low-cost features, face the issue of random shape/thickness of the resulting films from the mechanical strategy and the diminished semiconductor properties of the films. Therefore, they are unsuitable for the production of 2D TMD materials for large areas over wafer-scale and high-throughput applications. In this context, the wet-chemical-based method seems to be the method of choice for synthesizing high-quality MoS_2_ with a controllable number of layers in a relatively simple and easy way.

In the solution method, reports of jet-printed MoS_2_-based TFTs with printed source and drain (S/D) electrodes are still rare. So far, there have been several metal nanoparticle pastes that can be printed, such as nanoparticle pastes of Au, Al, Cu, Ag, etc. Additionally, gold is a highly conductive material, but its prohibitive price is disadvantageous for use in mass production of TFT_S_. In contrast, aluminum and copper are preferred because of their low price and good conductivity, but they are easily oxidized in the atmosphere, resulting in the degradation of their electrical features. Meanwhile, silver shows many outstanding merits, such as having the highest electrical conductivity among the materials that can be printed, along with its chemical stability and affordable price, making Ag stand out from the other materials. The undeniable possibility of using patterned Ag for electrodes in TFT devices fabricated by EHD jet printing was confirmed by our previous research [10]. In this previous work, the sheet resistance of printed Ag was evaluated to be around 0.027 Ω^−1^—comparable or even superior to that of Ag layers made by other methods, such as inkjet printing and screen printing [11,12]. Hence, we chose high-viscosity Ag paste for patterning the source and drain for the TFTs in this work. 

**Figure 1:**
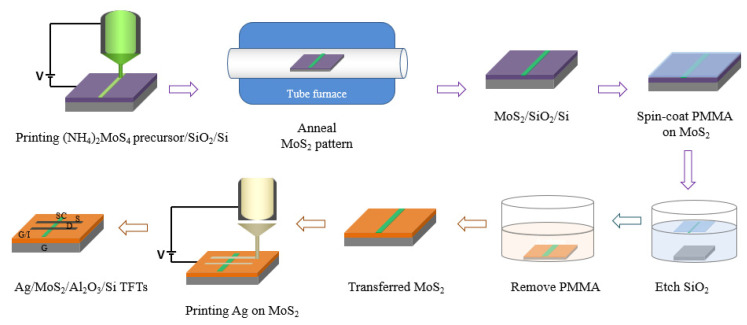
The whole process of fabricating TFT devices, including the EHD jet printing of the MoS_2_ semiconductor, the PMMA-based transfer of MoS_2_ onto Al_2_O_3_/Si, and the EHD jet printing of Ag as the S/D of the TFTs.

The improvement of TFTs’ characteristics by using a high-k dielectric has been studied previously [13]. However, the use of an Al_2_O_3_ dielectric layer in solution-based MoS_2_ TFTs with printed Ag S/D contacts—especially in a back-gated configuration—has not been explored to date. Hence, this work presents the advantages of EHD jet-printing technology in patterning electrical elements from various viscous materials and investigates the properties of high-k dielectric-based solution-processed MoS_2_ TFTs (Figure 1). This work demonstrates the suitability of direct EHD jet-printing technology for mass production of TFTs because of its low cost and high performance.

## 2. Experimental Details

### 2.1. Growth of MoS_2_ Layers

MoS_2_ patterns were created by a combination of EHD jet printing and one-step annealing following the process described in our previous research [9]. First, the ammonium tetrathiomolybdate ((NH_4_)_2_MoS_4_) precursor solution for printing was prepared with concentrations of 25, 50, 75, and 100 mM by stirring the (NH_4_)_2_MoS_4_ in a group of solvents of ethanolamine and butylamine for 12 h. Subsequently, a S-rich solution was formed by preparing a 1 M sulfur solution with carbon disulfide (CS_2_) and dissolving the sulfur solution in the (NH_4_)_2_MoS_4_ precursor solution with N,N-dimethylformamide. All chemicals used in this formulation were purchased from Sigma-Aldrich (Milwaukee, WI, USA) and ThermoFisher (Fisher Scientific, Leicestershire, UK) and used without further purification. 

For the printing of (NH_4_)_2_MoS_4_ on a UV/O_3_-irradiated 300 nm thick SiO_2_/Si substrate, the as-prepared precursor solution was collected in a syringe pump connected to a vertically movable plastic tip. The target substrate was then placed stably on a metal stage that could be moved on a horizontal plane, as described in our previous report [10]. Subsequently, for patterning the (NH_4_)_2_MoS_4_ lines, the voltage was adjusted to stretch the meniscus of the solution at the tip’s mouth into an upside-down cone shape, called the Taylor cone-jet mode. In particular, the printing parameters for patterning the (NH_4_)_2_MoS_4_ lines were a tip height of 2 mm, an applied voltage of 1.8–1.9 kV, a substrate temperature of 50 °C, a solution flow rate of 0.0032 μL s^−1^, and a stage speed range of 2000–8000 μm s^−1^. After printing the line patterns from the S-rich (NH_4_)_2_MoS_4_ solution, the patterns were pre-annealed at 150 °C for 20 min in ambient air using a hot plate. The pre-annealed patterns were then moved into a tube furnace for their annealing at a high temperature of 1000 °C for 1 h in a low vacuum (10^−1^–10^−2^ Torr), without sulfurization or further post-annealing, resulting in the final crystalline MoS_2_ line patterns. 

### 2.2. Transfer of MoS_2_ onto Other Substrates

The printed MoS_2_ on the SiO_2_/Si substrate, after annealing, was covered with a PMMA (avg. Mol wt. ~350,000 g mol^−1^ and ~996,000 g mol^−1^) layer using spin-coating (at a spinning speed of 3000 rpm for 30 s). The spin-coated sample was then baked at 200 °C for 2 h on a hot plate in ambient air. Subsequently, the PMMA/MoS_2_/SiO_2_/Si wafer was placed on the surface of an etchant mixture (HF:BOE:DI water (1:1:1)) to remove the SiO_2_. The resulting PMMA/MoS_2_ membrane was then cleaned from the etchant solution using deionized (DI) water. After discarding the etchant contaminant, the PMMA/MoS_2_ double layer was picked up on different target substrates for different purposes—for example, on Al_2_O_3_/Si for TFT fabrication, or on a Cu grid for transmission electron microscopy (TEM, Ultra-Corrected-Energy-Filtered -TEM Libra 200 HT Mc Cs) studies. Finally, the top PMMA layer was removed using acetone at 80 °C to obtain the patterned MoS_2_. 

### 2.3. Device Fabrication

Printed TFTs were fabricated by EHD jet printing with MoS_2_ line patterns as the semiconductor layer and Ag line patterns as S/D electrodes in each TFT. For a bottom gate and top contact (BGTC) configuration of the TFT, first, 40 nm thick alumina was deposited by atomic layer deposition (ALD) on clean bare Si wafers. The as-grown/printed MoS_2_ was then transferred carefully from the SiO_2_/Si substrate onto a cleansed Al_2_O_3_/Si substrate using the above procedure involving PMMA. Notably, the smooth morphological surface of the MoS_2_ transferred onto other substrates was shown using an atomic force microscope (AFM, Nano expert II EM4SYS) to have no wrinkles or damage [14]. Finally, linear Ag patterns/terminals were printed perpendicularly on the MoS_2_ line patterns with the assistance of the pneumatic pressure due to the high viscosity of Ag. In particular, the silver line patterns were successfully EHD-printed with a tip–substrate gap of 1.5 mm, applied voltage of 1 kV, stage speed of 2000–2500 μm s^−1^, and pressure of 80 kPa, and they were sintered at 200 °C for 30 min in ambient air. The whole fabrication process, from the preparation of the MoS_2_ line patterns to their transfer and the fabrication of the TFT devices, is sketched out in Figure 1.

## 3. Results and Discussion 

### 3.1. Printed MoS_2_ Line Patterns

**Figure 2:**
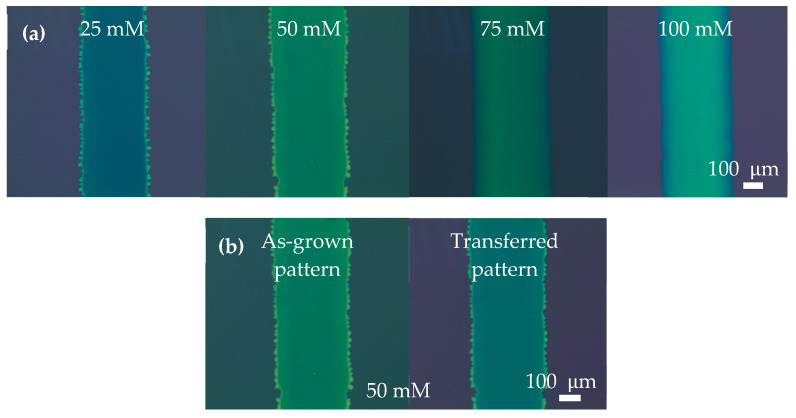
(**a**) Optical images of printed (NH_4_)_2_MoS_4_ linear patterns with four different concentrations. (**b**) Optical images of the same pattern of MoS_2_ after thermal annealing of as-grown/printed (NH_4_)_2_MoS_4_ and transfer onto SiO_2_/Si substrates, respectively.

Figure 2a shows the microscopic images of the MoS_2_ line patterns prepared with different (NH_4_)_2_MoS_4_ precursor concentrations using EHD jet printing. Visually, from left to right in this figure, the printed line patterns originating from increasing solution concentrations possess different thicknesses, which could be predicted from the color of the patterns. Moreover, all patterns showed smooth surfaces that were hole-free regardless of the concentrations (from 25 to 100 mM), demonstrating the printability and appropriately chosen concentration of the precursor solution. In addition, based on Figure 2b, the MoS_2_ pattern was proven to be undamaged and without wrinkles after transferring it using the PMMA-assisted method. Therefore, it is guaranteed that the MoS_2_ quality will be unchanged when the MoS_2_ is transferred to an arbitrary substrate to be used in further device fabrication.

**Figure 3:**
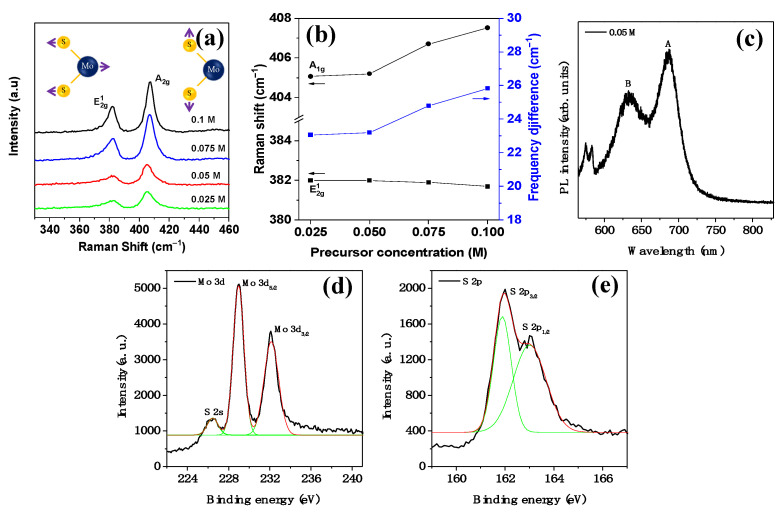
(**a**) Raman spectra and (**b**) frequencies of the $A_{1g\;}$and $E_{2g}^{1}\;$ modes against the precursor concentration. (**c**) PL spectra and (**d,e**) XPS spectra of the printed MoS_2_ line patterns printed from the 50 mM concentration precursor solution.

For the evaluation of the composition and thickness of the printed MoS_2_, three methods of analysis—Raman, photoluminescence (PL), and XPS spectroscopies—were carried out on the printed MoS_2_. In particular, Raman spectroscopy was carried out under four different precursor solutions, from 25 to 100 mM. As shown in Figure 3a, two strong signals for the $E_{2g}^{1}$ (at around 380 cm^−1^) and $A_{1g}$ (at around 405 cm^−1^) modes emerged in the Raman spectra regardless of the molar concentrations of the precursor solution. These two major modes assigned to the in-plane vibration of the Mo and S atoms and the out-of-plane vibration of the S atoms provided good evidence for the presence of the 2H phase of MoS_2_. It was also found that the two modes exhibited a well-defined concentration dependence, with the modes shifting opposite to one another with increasing concentration. Indeed, as the concentration was increased from 25 to 100 mM, the frequency difference between the modes increased gradually from 23.07 to 25.82 cm^−1^ (Figure 3b). Moreover, the Raman spectra suggested that the MoS_2_ line patterns obtained from concentrations of 25 mM and higher each consisted of at least three or four layers. Figure 3c describes the PL spectra of a representative MoS_2_ sample printed from the 50 mM precursor solution. Two specific peaks at about 686 and 632 nm in the spectra were attributed to the A_1_ and B_1_ excitons originating from the transition at the K-point of the Brillouin zone in the sample material, respectively.

The chemical composition of the printed MoS_2_ was determined by XPS. Figure 3d,e show the Mo 3d and S 2p regions, respectively, for the MoS_2_ that was printed from the 50 mM precursor solution and annealed. The Mo 3d XPS spectra were clearly observed to have two distinct peaks at 229.0 and 232.1 eV and a weak peak at 226.4 eV corresponding to the 3d_5/2_ and 3d_3/2_ of Mo^4+^ and S 2s, respectively. These peaks proved the presence of the 2H phase in MoS_2_. In addition, the peaks observed at 162.1 and 163.4 eV belonged to the divalent sulfide ions (S^2−^) 2p_3/2_ and 2p_1/2_ in 2H-MoS_2_, respectively. 

**Figure 4:**
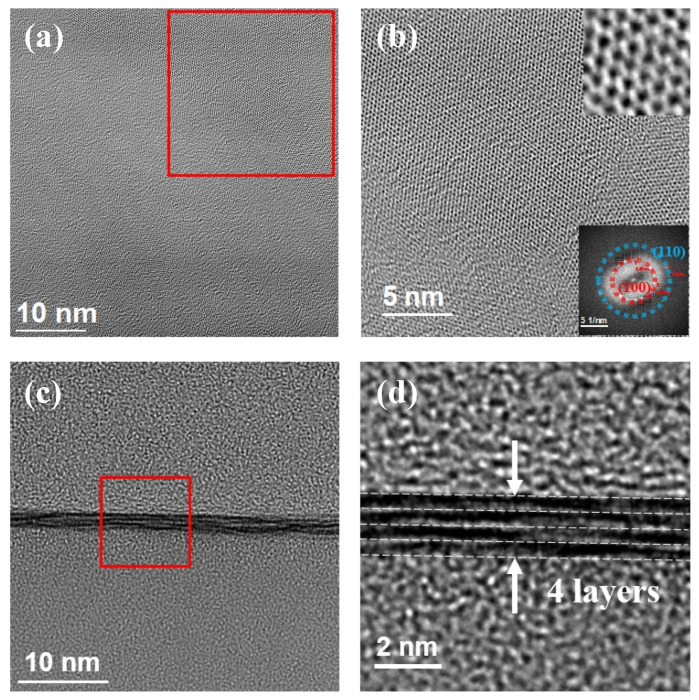
MoS_2_ line patterns for TEM printed from the 25 mM precursor solution: (**a**) Plan-view TEM image and (**b**) HR-TEM image of the selected MoS_2_ surface in (**a**), and the upper and lower inserts of (**b**) are the magnification of a portion of (**b**) and the corresponding fast Fourier transform, respectively. (**c**) Cross-sectional view of the TEM image and (**d**) the magnified image of the selected area in (**c**) containing the printed 4-layer MoS_2_ line patterns.

Furthermore, typical nanoscale images of the MoS_2_ line pattern printed from the 25 mM concentration precursor solution were captured by TEM to observe the plane-view and thickness of the pattern. Figure 4a,b show the morphological TEM images of the printed line pattern at low and high resolutions of the TEM, respectively, revealing the honeycomb MoS_2_ surface. Moreover, this feature can be clearly seen in the top insert of Figure 4b. Furthermore, the fast Fourier transform (FFT) corresponding to the lower insert of Figure 4b depicts the polycrystallization of the printed MoS_2_. The layer number of the line pattern was found to be four monolayers from the cross-sectional view of the TEM image (Figure 4c). Meanwhile, the selected area of Figure 4c, magnified to the scale shown in Figure 4d, corresponded to the tetra-layer of the pattern.

### 3.2. Printed Ag Line Patterns 

**Figure 5:**
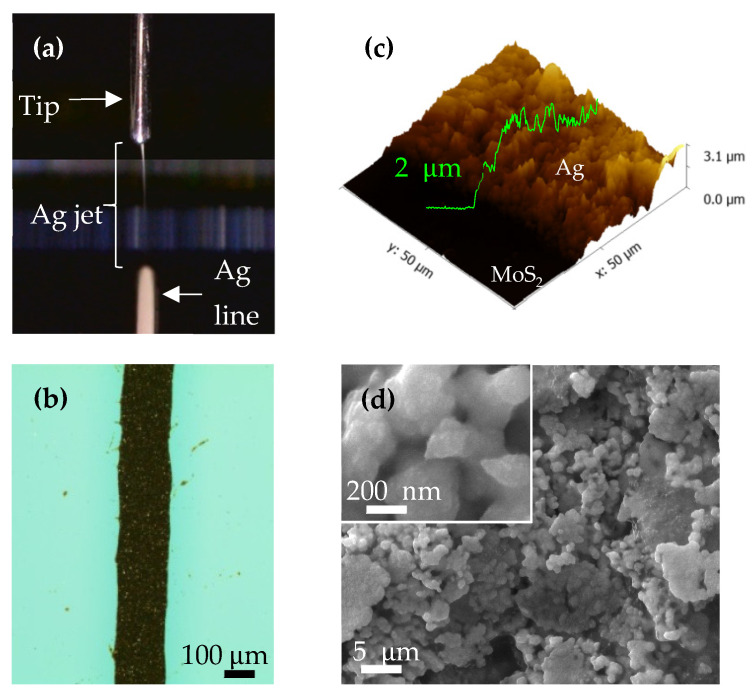
(**a**) Photograph of the Taylor cone-jet mode used for printing the Ag line patterns. (**b**) Optical image of a printed Ag line pattern. (**c**) AFM image at the boundary area including the Ag and MoS_2_ line patterns, showing the thickness of the printed Ag line pattern. (**d**) SEM images of the printed Ag line pattern after sintering.

The printing process of the Ag line patterns, illustrated in Figure 5a, clearly shows that the Taylor cone-jet mode was used to print the pattern. The width and clear shape of the Ag line patterns were found to be strongly dependent on the printing parameters—such as pressure, additive existence, and especially the stage speed—in our previous research [10]. Here, Figure 5b shows a typical microscope image of a Ag line pattern printed under the optimized conditions previously mentioned in the experimental section. The patterns were each observed to be 100–200 μm in width, without serrated edges. Figure 5c shows the AFM height step image of the printed Ag line pattern on MoS_2_, and the thickness of the pattern was measured to be 2 μm. Finally, according to the SEM image of the printed Ag line pattern after sintering, as shown in Figure 5d, although a rough morphology of the pattern was seen in the image, the distribution of the Ag particles was found to cover the entire substrate surface.

### 3.3. Printed MoS_2_ TFTs

**Figure 6:**
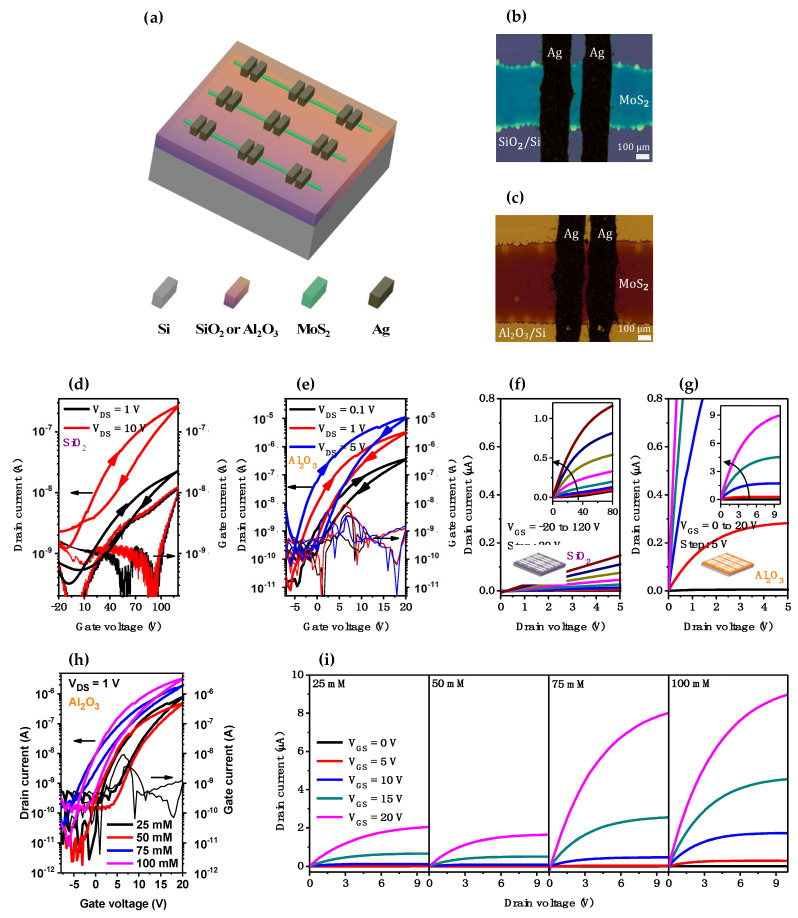
(**a**) Diagram of cross-sectional TFTs. (**b**,**c**) Top-view optical images of real printed MoS_2_ TFTs based on SiO_2_ and Al_2_O_3_, respectively, with Ag S/D electrodes. (**d**,**e**) Transfer curves and (**f**,**g**) output curves of MoS_2_ TFTs based on SiO_2_ (V_GS_: −20 to 120 V) and Al_2_O_3_ (V_GS_: 0 to 20 V), respectively, fabricated from the same 100 mM solution. (**h**,**i**) Transfer and output curves of Al_2_O_3_-based MoS_2_ TFTs fabricated from 4 different concentrated precursor solutions.

The electrical properties of the printed MoS_2_ were characterized in a thin-film transistor application in which the integration of an ALD 40 nm Al_2_O_3_ dielectric, a printed MoS_2_ line pattern as a semiconductor, and printed Ag line patterns as top contacts was carried out. In this study, a counterpart TFT based on low-k SiO_2_ was also fabricated for comparison with the aforementioned high-k Al_2_O_3_-based TFT. Figure 6a shows the schematic of the BGTC MoS_2_ TFTs. Additionally, the top-view optical images of two typical TFTs, in which MoS_2_ was grown on SiO_2_ (k = 3.9) and transferred onto Al_2_O_3_ (k = 7.0), respectively, are shown in Figure 6b,c, respectively. 

The field-effect mobility (μ) and threshold voltage ($V_{\mathrm{TH}}$) were calculated in the linear regime at a drain voltage ($V_{\mathrm{DS}}$) of 1 V for the TFT. The threshold voltage is defined as the intersection point of the $V_{\mathrm{GS}}$ axis and the extrapolation of the linear region of the transfer curve. The linear field-effect mobility from each device was then calculated from the gradient of the drain current versus the gate voltage according to the following equation:

(1) $$\mu =\left.\frac{L}{{\mathrm{WCV}}_{\mathrm{DS}}}\times \frac{{\mathrm{dI}}_{\mathrm{DS}}}{{\mathrm{dV}}_{\mathrm{GS}}}\right|{\;}_{V_{\mathrm{DS}}=1\;V}$$

where L and W are the patterned MoS_2_ channel length and width, respectively, and C is the capacitance per unit area of the gate insulator. The length and width of the patterned MoS_2_ channel were about 40–100 and 500–600 μm (for various devices), respectively, used for calculating the TFTs’ performance in relation to the precursor concentrations and the dielectric layers, respectively. 

For the primary evaluation of the TFTs, two TFT device groups with different dielectrics of Al_2_O_3_ (15.5 × 10^−8^ F cm^−2^) and SiO_2_ (10^−8^ F cm^−2^) were prepared from the same 100 mM MoS_2_ precursor solution. The $I_{\mathrm{DS}}$ − $V_{\mathrm{GS}}$ transfer curves of these MoS_2_ devices are shown in Figure 6d,e. Due to the dissimilar materials and thicknesses of the dielectric layer, the applied voltage range should be different to prevent the breakdown of the devices. While sweeping $V_{\mathrm{GS}}$ from −20 to 120 V (and back) at various constant $V_{\mathrm{DS}}\;$values of at least 1 V for the 300 nm SiO_2_ TFT (Figure 6d), those for 40 nm Al_2_O_3_ were measured at about −10 V to 20 V (then back) and 0.1 V (Figure 6e), respectively. Notably, both TFT device types showed a clockwise hysteretic phenomenon associated with the electron trap at the MoS_2_–dielectric and/or MoS_2_–Ag interfaces. However, a dramatic reduction in hysteresis of one order of magnitude was achieved when using Al_2_O_3_ instead of SiO_2_ in the TFT, revealing smaller trap charges at the interface between MoS_2_ and Al_2_O_3_. In addition, although the maximum gate leakage current was about 10^−9^–10^−8^ A for the low- and high-dielectric-based TFT devices, a current ($I_{\mathrm{DS}}$) two orders of magnitude higher was obtained when using Al_2_O_3_ instead of SiO_2_ as the gate insulator. Simultaneously, the on/off current ratio ($I_{\mathrm{on}}/I_{\mathrm{off}}$) of each device with Al_2_O_3_ (~10^5^) was obviously enhanced compared to that of each device with SiO_2_ (~10^2^). Moreover, other electrical parameters of each MoS_2_ TFTs showed a remarkable improvement after changing the dielectric from low-k SiO_2_ to high-k Al_2_O_3_, such as a 30 times steeper subthreshold swing (SS), a negatively shifted threshold voltage, and an 80 times increased carrier mobility. Furthermore, all devices exhibited an n-channel transistor. This was consistent with the $I_{\mathrm{DS}}$ − $V_{\mathrm{DS}}$ output characteristics shown in Figure 6f,g. The output curves were linear in the low-bias range ($V_{\mathrm{DS}}\;$< 20 and 2 V for SiO_2_ and Al_2_O_3_, respectively) and saturated in the higher drain bias region.

Having confirmed the advantages of using Al_2_O_3_ as the dielectric, MoS_2_ TFT devices were fabricated with different patterned MoS_2_ channel thicknesses from different precursor concentrations. Figure S1 shows the hysteresis of the gate transfer and output characteristics of MoS_2_/SiO_2_ TFTs, among which the best performance belonged to the devices fabricated from the 50 mM precursor solution. The highlightable electrical properties of these 50 mM MoS_2_ devices were an on/off current ratio of ~10^4^ and a mobility of 0.024 cm^2^ V^−1^ s^−1^ (Table S1), which were two and one order of magnitude increases compared to those of the devices with a thicker MoS_2_ layer, respectively. Moreover, as with the Al_2_O_3_-based TFTs, the hysteresis became smaller with a thicker MoS_2_. This thickness-dependent behavior indicates that the surface of the MoS_2_ plays a crucial role in hysteresis [15]. 

In general, each MoS_2_ TFT with a SiO_2_ dielectric possessed fluctuated and much lower features than its counterpart with an Al_2_O_3_ layer. Meanwhile, the MoS_2_/Al_2_O_3_ devices’ performance was commonly more stable under different precursor concentrations (Figure 6h). In addition, the fully saturated output curves obtained for each Al_2_O_3_-based TFTs were better than those obtained for the corresponding SiO_2_-based TFTs that lacked a saturation region with an excessively thick MoS_2_ layer. (Figure 6i and Figure S1d–f).

The respective characteristics of all TFT devices with Al_2_O_3_ are summarized in Table 1. In particular, the Al_2_O_3_-based TFTs exhibited optimal properties of μ ≈ 0.9 cm^2^ V^−1^s^−1^, SS ≈ 1.0 V dec^−1^, $\frac{I_{\mathrm{on}}}{I_{\mathrm{off}}}\;\approx$ 5 × 10^5^, and $V_{\mathrm{TH}}$ ≈  7.0 V, comparable to the properties reported in some previous solution-based works [16,17,18]. On the other hand, it was also noted that the field-effect mobility of each of our devices was lower than that of devices using different synthesis methods of MoS_2_, such as the CVD methods [19] or other S/D electrodes [20]. This lower mobility was a result of the following factors stemming from using the printed Ag line pattern: (1) a rough interfacial contact between Ag and MoS_2_, (2) an imperfectly clean area between the S/D electrodes due to the Ag printing process, and (3) a different barrier of the channel layer and S/D electrodes. Eventually, the result of this work confirmed that Al_2_O_3_ and Ag could be used as dielectric and gate electrode materials for MoS_2_ TFTs, respectively. In essence, using Al_2_O_3_ dielectrics instead of SiO_2_ can improve the mobility, current ratio, S-S factor, and hysteresis of TFT devices based on them, due to the screening effect of the dielectric on carrier scattering. 

## 4. Conclusions

We demonstrated that an EHD jet printer could be used for multi-printing of a MoS_2_ semiconductor and Ag electrodes for TFT fabrication. The MoS_2_ pattern was obtained from the printed precursor solution after simple annealing. The MoS_2_ films proved to be undamaged without wrinkles after transferring them to another substrate. When employing a high-k gate dielectric, all electrical properties of the TFTs could be improved due to the screening effect of the dielectric on carrier scattering. A controllable hysteretic behavior achieved by varying the MoS_2_ thickness and the dielectric materials showed the potential for electronic device applications. Concurrently, the application of a stable printing technique for 2D materials for the synthesis of semiconductors and commercial pastes for the fabrication of electrodes is a fundamental building block towards low-cost and enhanced-performance electronics on a large scale.

## Figures and Tables

**Table 1:** Respective characteristics of the Al_2_O_3_-based printed MoS_2_ TFTs.

Gate Dielectric	Concentration mM	$I_{\mathrm{on}}/I_{\mathrm{off}}\;$	S-S (V dec^−1^)	$V_{\mathrm{TH}}$ (V)	μ (cm^2^ V^−1^ s^−1^)	Hysteresis (V)
40 nm Al_2_O_3_	25	(2.1 ± 1.7) × 10^5^	2.2 ± 0.9	13.1 ± 2.4	0.29 ± 0.23	4.0 ± 0.65
	50	(2.7 ± 1.7) × 10^5^	1.3 ± 0.4	11.0 ± 1.6	0.33 ± 0.06	4.1 ± 0.15
	75	(7.5 ± 2.1) × 10^4^	4.1 ± 0.9	10.1 ± 1.5	0.42 ± 0.14	2.5 ± 0.23
	100	(1.7 ± 1.1) × 10^5^	2.5 ± 0.5	9.5 ± 2.9	0.58 ± 0.3	3.2 ± 0.57

## Data Availability

The data are available from the corresponding authors upon reasonable request.

## Supplementary Materials

The following supporting information can be downloaded at: https://www.mdpi.com/article/10.3390/nano13010194/s1, Figure S1: (a–c) Transfer characteristic curves with hysteresis behavior and (d–f) output characteristic curves of SiO_2_-based MoS_2_ TFTs prepared from 50 mM, 75 mM and 100 mM solution concentrations.; Table S1: Characteristics of the SiO_3_-based printed MoS_2_ TFTs.
