# Supplementary material for: Improved Electrical Properties of EHD Jet-Patterned MoS2 Thin-Film Transistors with Printed Ag Electrodes on a High-k Dielectric
Source: Nanomaterials (Basel). 2023 Jan 1;13(1):194. doi: 10.3390/nano13010194 (PMC9824249; doi:10.3390/nano13010194)
Supplement: Supplementary file 1 [file nanomaterials-13-00194-s001.zip › nanomaterials-2077593-supplementary.pdf]

Supplementary Information

Improved Electrical Properties of EHD Jet-Patterned MoS_2 Thin-Film Transistors with Printed Ag Electrodes on a High-k Dielectric

Thi Thu Thuy Can † and Woon-Seop Choi *

School of Electronics and Display Engineering, Hoseo University, Asan 31499, Republic of Korea;
*Correspondence: wschoi@hoseo.edu
†Current address: Faculty of Physics, Hanoi National University of Education, Hanoi, Vietnam

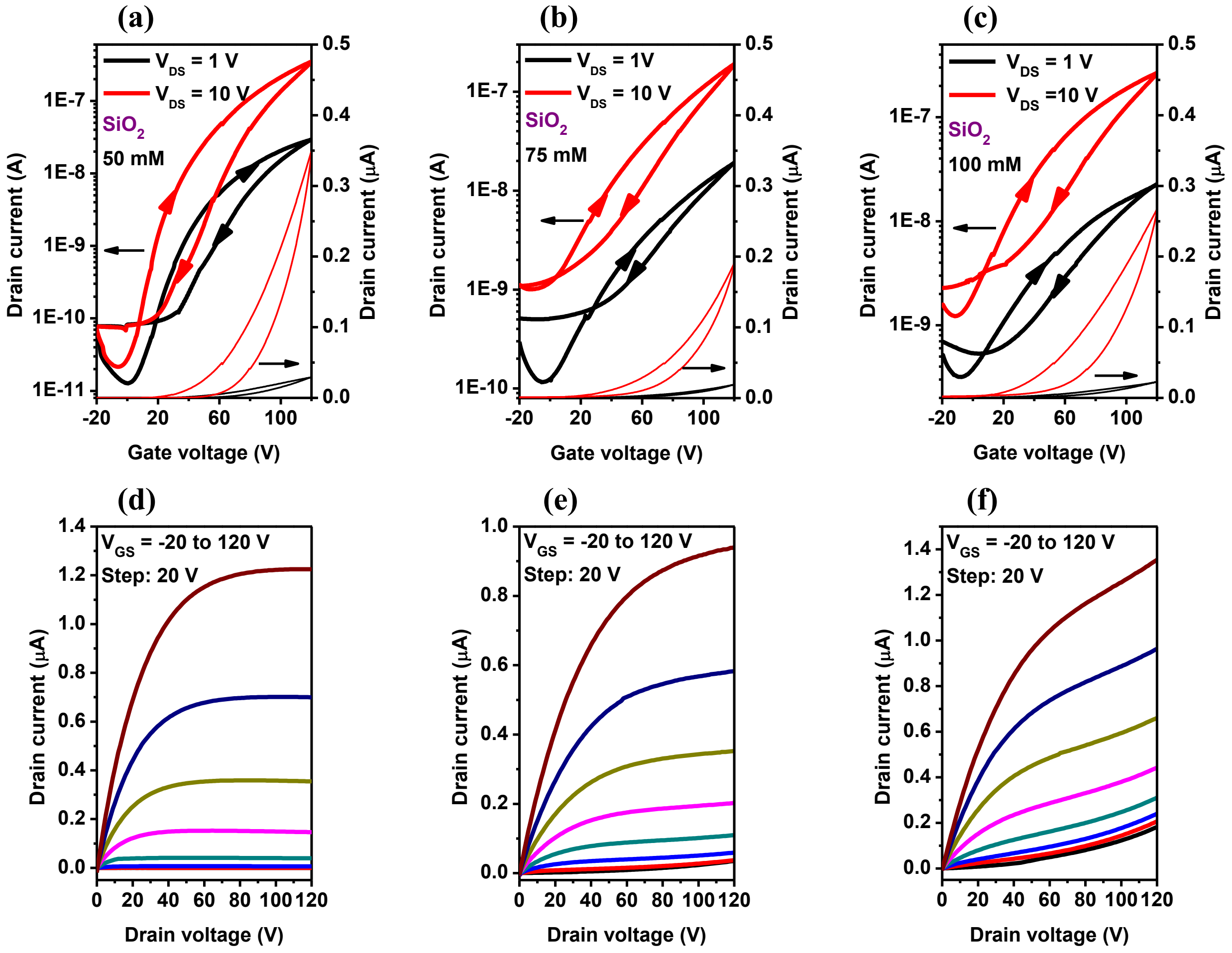

Figure S1. (a-c) Transfer characteristic curves with hysteresis behavior and (d-f) output characteristic curves of SiO_2-based MoS_2 TFTs prepared from 50 mM, 75 mM and 100 mM solution concentrations.

Table S1 Characteristics of the SiO_2-based printed MoS_2 TFTs.

G/I	Concentration [mM]	I_{on}/I_{off}	S-S [V dec^{-1}]	μ [cm^2 V^{-1} s^{-1}]	Hysteresis [V]
SiO_2	50	(1.2±0.8)×10^4	15.5±4.4	0.024±0.02	29.3±4.9
	75	(9.0±5.1)×10^1	53.2±10.3	0.0036±0.0013	17.6±3.6
	100	(9.6±5.5)×10^1	53±12	0.0072±0.0017	20.3±4.9
